# Development of a UPLC-MRM-based targeted proteomic method to profile subcellular organelle marker proteins from human liver tissues

**DOI:** 10.1038/s41598-022-15171-0

**Published:** 2022-06-29

**Authors:** Xiazi Qiu, Laura M. Doyle, Michael Zhuo Wang

**Affiliations:** Department of Pharmaceutical Chemistry, School of Pharmacy, University of Kansas, Lawrence, KS USA

**Keywords:** Analytical biochemistry, Liquid chromatography, Mass spectrometry, Organelles, Biomarkers

## Abstract

Subcellular organelles have long been an interest in biochemical research and drug development as the isolation of those organelles can help to probe protein functions and elucidate drug disposition within the cell. Usually, the purity of isolated subcellular organelle fractions was determined using immunoblot analysis of subcellular organelle marker proteins, which can be labor-intensive and lack reproducibility due to antibody batch-to-batch variability. As such, a higher throughput and more robust method is needed. Here, a UPLC-MRM-based targeted proteomic method was developed for a panel of human organelle marker proteins and used to profile a series of sucrose fractions isolated from the protein extract of human liver tissues. The method was validated by comparing to the traditional immunoblot and determining subcellular localization of three case study proteins (CYP3A4, FcRn, and β2M) pertaining to the disposition of small molecule and biologic drugs. All three case study proteins were co-enriched with their corresponding subcellular protein marker, and complete recoveries were achieved from isolated fractions. This newly developed MRM method for the panel of human organelle marker proteins can potentially accelerate future intracellular drug disposition analysis and facilitate subcellular organelle quality assessment.

## Introduction

Subcellular organelles are compartmentalized lipid membrane-surrounded structures within a eukaryotic cell, and they carry out specific biological functions. They include mitochondria for energy production, endoplasmic reticulum (ER) and Golgi apparatus for protein and lipid synthesis and processing, and lysosome for protein degradation and recycling. Because of the importance of organelles in cell biology, these subcellular structures have been studied and probed using various methods. These methods include immunocytochemistry, which utilize antibodies of a specific organelle marker protein or fluorescently labeled proteins, to study the morphology of organelles or the co-localization of a target component in situ. Another approach is to separate and purify each subcellular organelle using different fractionation methods (e.g., rate zonal centrifugation and differential centrifugation). The purity of each fraction can then be characterized using immunoblot analysis to probe the distribution of organelle marker proteins, or sometimes, by measuring the activity of marker enzymes. In general, these characterization methods either require specific antibodies for each of the target proteins or availability of a marker substrate reaction.

Research in subcellular organelles has seen a rise towards global profiling analysis facilitated by the development of modern mass spectrometry (MS) and MS-based proteomics. Recent MS analyses of various organelles isolated from different species were comprehensively reviewed by Yan et al.^1^. Such global proteomics allowed the identification of previously-unknown proteins that functionally-associated with specific organelles. Andreyev et al. used global proteomics to screen and identify unique, universal and unbiased protein marker ensembles for general organelles, which allowed more precise and accurate assignment of components of interest within the cell^2^. In addition to the global profiling, quantitative proteomic analysis of organelles can be used to study the dynamics of protein translocation among different states by profiling their abundance within various subcellular organelles^1,3^. Mann et al. introduced the concept of protein correlation profiling (PCP), using quantitative proteomics to study the centrosome composition^4^, and to provide a means to categorize and map the mouse liver proteins among subcellular organelles^5^. For such global proteomic analysis, data are usually biased towards moderate-to-high abundance proteins if no depletion strategy is employed, and low abundance proteins are often missed^6^.

In contrast to global proteomics, targeted proteomics is a robust, sensitive, and specific method for protein quantification in complex biological samples^7–10^. Targeted proteomics is often operated under multiple-reaction-monitoring (MRM) mode and utilizes unique signature peptides as surrogates for the protein of interest. By only detecting ions that have the pre-selected precursor and product mass-to-charge ratios, MRM is capable of detecting and quantifying multiple proteins with high specificity. This allows analysis of different subcellular organelle markers within the same LC–MS run. However, despite such high potential for multiplexed organelle marker analysis, such a marker MRM panel has not been developed or cross-validated with immunoblot previously.

Therefore, the objective for this study is to develop ultra-performance liquid chromatography (UPLC)-MRM-based targeted proteomic methods for a subcellular organelle marker panel, and to demonstrate that the MRM-based targeted proteomic method is a suitable alternative to traditional antibody-based subcellular organelle profiling. Furthermore, the subcellular localization of three case study proteins, which are important to the disposition of small molecule and biologic drugs, including an endosome-enriched protein (FcRn and its binding partner β2M) and an ER protein (CYP3A4), will be determined to validate the developed method as a new way to study the subcellular distribution of protein targets recovered from a gradient fractionation.

## Materials and methods

### Chemical reagents

Ammonium bicarbonate, dithiothreitol, trichloroacetic acid solution (6.1N), iodoacetamide, sodium deoxycholate, and sucrose were obtained from Sigma-Aldrich (St. Louis, MO). Sodium chloride, Optima grade acetone, Optima LC/MS grade water, acetonitrile, water with 0.1% (v/v) formic acid, and formic acid were purchased from Fisher Scientific (Fair Lawn, NJ). Imidazole and phenylmethylsulfonyl fluoride (PMSF) were from Acros Organics (NJ, USA), and EDTA was from Fluka Biochemika (Buchs, Switzerland). Anhydrous 2-propanol was acquired from Alfa Aesar (Ward Hill, MA). Bicinchoninic acid assay (BCA) and albumin standard were from Thermo Scientific (Rockford, IL). Frozen sequencing grade modified trypsin was purchased from Promega (Madison, WI). Synthetic ^13^C and ^15^N stable isotope-labeled (SIL heavy peptides) crude signature peptides were manufactured by ThermoFisher Scientific (> 99% isotopic enrichment according to the manufacturer) and were delivered in 50% acetonitrile and 0.1% trifluoroacetic acid.

### Human liver tissues

Three human donor liver tissue samples (Supplemental Table 1) used for this study were acquired from the University of Kansas Liver Center Tissue Bank (Kansas City, Kansas). These livers were collected under protocols approved by the Institutional Review Board of the University of Kansas Medical Center.

### Subcellular fractionation using sucrose gradient

Post-nuclear supernatants (PNS) from the three donor liver tissues were collected following the published protocol^7^, and 400 μL PNS was diluted with 100 μL homogenization buffer (3 mM imidazole, 1 mM EDTA, and 250 mM sucrose, pH 7.4), and was layered on top of a step sucrose gradient, which consisted of 3 mL 25% (w/w) sucrose, 4.5 mL 35% (w/w) sucrose, and 3 mL 40% (w/w) sucrose in an ultra-clear centrifuge tube (Beckman Coulter, Brea, CA). All sucrose solutions were prepared in 3 mM imidazole and 1 mM EDTA (pH 7.4). The centrifugation was carried out in a SW41. Ti rotor using an Optima L-90 K ultracentrifuge (Beckman Coulter, Brea, CA) at 24,200 rpm and 4 °C for 3 h with slow acceleration and no brake deceleration. Fractions (12 total) were taken by pipetting 1 mL from the top to the bottom (fraction #1 through #12), except for the first and the last fractions that contained 500 μL. Total protein concentrations were determined for the PNS and each isolated fraction using the BCA assay following the manufacturer’s protocol. For PNS and sucrose fractions #1 and #2, the diluent used for the BCA assay was homogenization buffer; for the rest of the collected sucrose fractions, the diluent used for the BCA standards was 25% sucrose solution containing 3 mM imidazole and 1 mM EDTA (pH 7.4) to account for effect of sucrose on total protein quantification by the BCA assay.

### Immunoblot

PNS (2 μL) and sucrose fractions (5 μL) were separated on a 4–15% precast Tris-glycine SDS-PAGE gel (Bio-Rad Laboratories, Hercules, CA) under reducing conditions at 160 V for 1 h on ice. After separation, proteins were transferred onto PVDF membranes (Bio-Rad Laboratories) using a Bio-Rad Trans-Blot Turbo transfer system. Each membrane was blocked using 5% (w/v) non-fat milk in 1 × TBS-T (Tris-buffered saline with Tween 20, 0.1% v/v) at room temperature for 2 h. After washing three times with 1× TBS-T, they were incubated with the corresponding primary antibody solutions (Supplemental Table 2, Cell Signaling Technologies, Danvers, MA) in 5% (w/v) bovine serum albumin (BSA) (Fisher Bioreagents, Fair Lawn, NJ) in 1× TBS-T at 4 °C overnight. After washing with 1× TBS-T three times, each membrane was then incubated with secondary antibody (Supplemental Table 2, Cell Signaling Technologies) at room temperature for 2 h. Following washing with 1× TBS-T three times, each membrane was probed with ClarityMax™ Western ECL substrate (Bio-Rad Laboratories) and imaged using Image Station 440 CF (Kodak Digital Science, Rochester, NY).

### Protein precipitation and on-pellet trypsin digestion

Sucrose fractions and PNS (20 μg) were diluted with water to a final volume of 100 μL or 1 mL (1 volume) depending on their total protein concentrations. Then, the samples were solubilized with 1/100th volume of 2% (w/v) sodium deoxycholate for 15 min at room temperature. After solubilization, 1/10th volume of trichloroacetic acid (100% w/v) was added, and the samples were incubated at 4 °C with gentle shaking overnight to achieve protein precipitation. After centrifugation at 16,000 × *g* at 4 °C for 15 min, the supernatant was discarded by aspiration using a pipette and the pellet was washed with 200 μL cold acetone (− 20 °C) by incubating on ice for 15 min. The pellet was collected after another centrifugation at 16,000 × *g* at 4 °C for 15 min and dried at 37 °C for three minutes. The resulting pellet was then re-solubilized with trypsin solution (1:20 w/w trypsin:protein) in 100 mM ammonium bicarbonate (pH 8.0) at 37 °C for four hours with gentle shaking. After resolubilization, trypsin digestion was carried out using the previously published protocol^7,9^.

### UPLC-MRM targeted proteomic methods

Signature tryptic peptides for each organelle marker protein were chosen following previously published criteria based on physicochemical properties, genetic polymorphism, and trypsin digestion efficiency^7,8,10^. Candidate signature peptides were further confirmed based on their MRM signal intensities observed during LC–MS analysis of SIL heavy peptides or by the product ion screening using trypsin digests of target proteins as described previously^8^. Although it was aimed to have at least two signature peptides for each target protein, only one signature peptide (Table 1) was confirmed for Rab5 (23.7 kDa) and Histone H3.1 (15.4 kDa) due to their small protein size. The single signature peptide used for CYP3A4 was previously developed and validated^10,11^. Synthetic stable heavy isotope labeled signature peptides were infused into a Waters Xevo TQ-S triple quadrupole MS coupled with a Waters Acquity I-class UPLC system (Milford, MA) for MRM transition development using IntelliStart (Waters). All other UPLC and MS parameters used were identical to what was previously published^7^. Briefly, the LC gradient consisted of (A) water with 0.1% formic acid and (B) acetonitrile with 0.1% formic acid and was run at 0.4 mL/min for 13.5 min total on a Waters UPLC BEH C18 analytical column (1.7 μm, 2.1 × 100 mm). The gradient started with 2% B for 1 min, then increased to 15% B for the next 2 min and to 30% B for the following 7 min. Column wash was achieved by running at 95% B for 1.5 min and re-equilibration was at 2% B for 2 min. Specific MRM transitions for signature peptides of organelle marker proteins and case study proteins are summarized in Table 1. SIL heavy peptides were spiked into all samples during quenching of trypsin digestion, and were used as internal standards to normalize different ionization efficiencies for the peptides from different LC–MS injections and confirm the identity of signature peptides by matching retention time. Both endogenous light peptides (released by trypsin from proteins in sucrose fractions) and spiked-in SIL heavy peptides transitions were monitored in each run and used for relative protein quantification (details in “Data analysis” section). No appreciable separation of SIL heavy peptides and corresponding light peptides were observed due to limited chromatographic resolution by the narrow bore analytical column used in our study.

**Table 1:** Signature peptides for organelle markers and three case study proteins and their corresponding MRM methods.

Protein	Signature peptide^a^	Start–end^b^	Average mass MH^+^ (Da)^c^	MRM (m/z)^d^		Cone (V)	CE^f^ (eV)
				Precursor ion	Product ion^e^		
β2M	IQVYSR	27–32	765.9	383.45	425.21	32	16
					**524.28**		12
					652.34		14
	IQVYS(R)		775.9	388.42	435.26		16
					**534.27**		12
					662.34		14
FcRn	LFLEAFK	97–103	868.1	434.54	365.22	20	18
					**607.35**		12
					754.41		16
	LFLEA(F)K		878.1	439.54	375.22		18
					**617.35**		12
					764.41		16
CYP3A4	EVTNFLR	244–250	879.0	440.10	**650.40**	40	10
	EVTN(F)LR		889.0	445.10	**660.40**		
HSC70	ITITNDK	501–507	804.92	402.73	477.20	40	16
					**590.30**		12
					691.40		14
	ITITND(K)		812.92	406.90	485.43		16
					**598.61**		12
					699.70		14
	FEELNADLFR	302–311	1254.39	627.30	435.30	40	28
					**735.40**		24
					848.50		20
	FEELNADLF(R)		1264.39	632.67	445.50		28
					**745.73**		24
					858.90		20
LAMP1	VWVQAFK	357–363	878.07	439.54	**592.35**	30	14
					778.42		16
	VWVQA(F)K		888.07	444.54	**602.35**		14
					788.42		16
	AFSVNIFK	349–356	926.11	463.56	294.18	30	22
					**707.41**		14
	AFSVNI(F)K		936.11	468.56	304.18		22
					**717.41**		14
	TVESITDIR	138–146	1034.16	517.58	704.39	30	18
					**833.44**		14
	TVESITDI(R)		1044.16	522.58	714.39		18
					**843.44**		14
HSP60	LSDGVAVLK	397–405	902.09	451.27	260.20	40	12
					430.30		13
					**701.42**		14
	LSDGVAVL(K)		910.09	455.48	268.31		12
					438.62		13
					**709.73**		14
	VTDALNATR	421–429	961.07	480.76	461.25	40	16
					645.37		16
					**760.39**		16
	VTDALNAT(R)		971.07	486.03	471.48		16
					655.66		16
					**770.78**		16
GRP78	LTPEEIER	533–540	987.10	493.76	546.29	40	18
					675.33		22
					**772.38**		18
	LTPEEIE(R)		997.10	499.03	556.56		18
					685.67		22
					**782.79**		18
	ITITNDQNR	524–532	1075.17	537.78	646.29	40	16
					**747.34**		18
					860.42		18
	ITITNDQN(R)		1085.17	543.10	656.63		16
					**757.72**		18
					870.83		18
Rab7	ATIGADFLTK	39–48	1037.21	519.11	432.74	40	14
					**751.40**		18
	ATIGADFLT(K)		1045.21	523.11	436.74		14
					**759.40**		18
	VIILGDSGVGK	11–21	1058.27	529.64	**619.30**	40	16
					845.47		16
	VIILGDSGVG(K)		1066.27	533.64	**627.30**		16
					853.47		16
Rab5	LVLLGESAVGK	23–33	1086.32	543.67	437.26	42	14
					**647.34**		16
	LVLLGESAVG(K)		1094.32	547.67	441.26		14
					**655.34**		16
Histone H3.1	YRPGTVALR	42–50	1033.23	517.12	320.17	42	22
					**713.43**		22
	YRPGTVAL(R)		1043.23	522.12	320.17		22
					**723.43**		22

^*a*^Stable isotope-labeled amino acid residues are included in parentheses.

^*b*^Start and end residue positions of peptides in the corresponding full-length protein.

^*c*^Theoretical average mass of mono-protonated molecular ion.

^*d*^All precursor ions have a charge state of + 2, and all product ions used as the quantification trace have a charge state of + 1.

^*e*^Bolded product ions were used as the quantification trace during MRM.

^*f*^CE = Collision Energy.

### Data analysis

The relative quantification of organelle markers and case study proteins were achieved using IS-normalized MRM peak areas by taking the ratio of peak areas between light signature peptides (released by trypsin from proteins in sucrose fractions) and spiked-in SIL heavy peptides using the TargetLynx software (Waters). Then the normalized response ratio was calculated by taking the ratio of IS-normalized MRM peak areas of a target protein between sucrose fractions and unfractionated PNS. Immunoblot band intensities were analyzed using Image J (Version 2.0.0-rc-43/1.52n, NIH)^12^. The normalized band intensity was calculated by taking the ratio of immunoblot band intensities of a target protein between sucrose fractions and unfractionated PNS. As such, both the normalized response ratio and the normalized band intensity can be used to indicate protein enrichment over PNS when the ratio is greater than 1. The average values and standard deviations obtained from three human liver donors were plotted. All data were processed and plotted using GraphPad Prism (version 8.4.3; San Diego, CA).

## Results

### Total protein concentrations and amount present in PNS and in each isolated sucrose fraction

**Figure 1:**
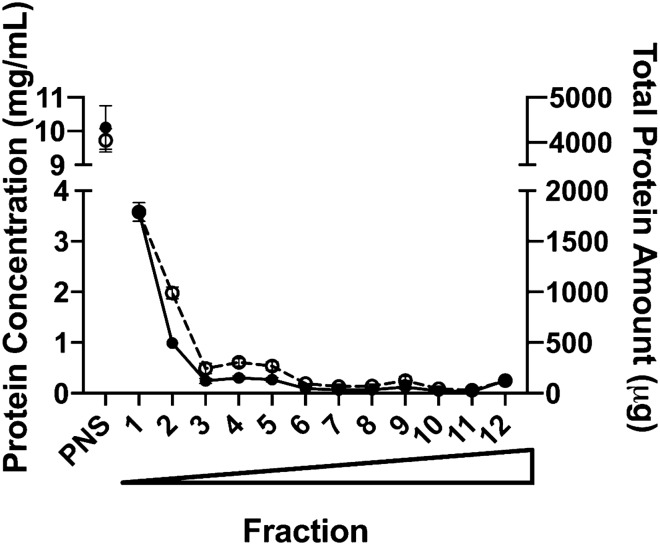
Protein concentration and total protein amount present in PNS and each isolated sucrose fractions. Solid circles represent protein concentration (mg/mL), and open circles represent total protein amount isolated (µg). Circles represent the average value from three human liver donors, and error bars represent the standard deviation. Wedge indicates increasing sucrose % from fraction 1–12.

Each isolated sucrose fraction and the PNS were subjected to BCA analysis to determine total protein concentrations and total protein amount present (Fig. 1). Total protein amount in each fraction was calculated based on the determined protein concentration and its isolated volume.

### UPLC-MRM methods for signature peptides of subcellular organelle markers and case study proteins

**Figure 2:**
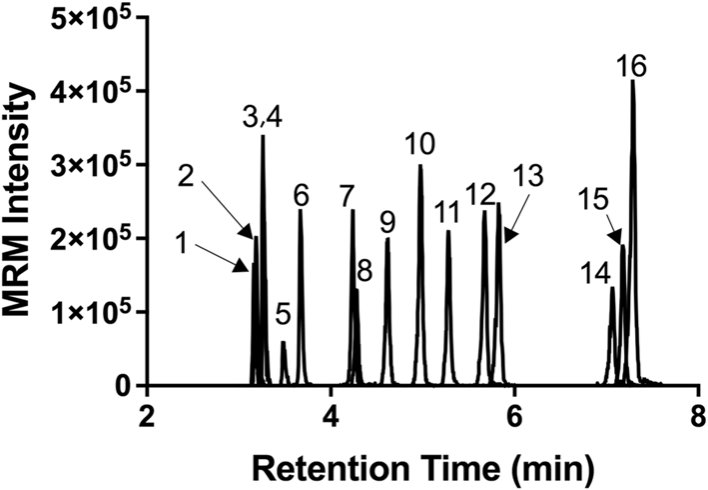
UPLC-MRM chromatograms of SIL heavy signature peptides for subcellular organelle marker proteins and three case study proteins. 1: HSC70_ITITNDK (Cytosol); 2: GRP78_ITITNDQNR (ER); 3: β2M_IQVYSR; 4: HSP60_VTDALNATR (Mitochondria); 5: HistoneH3.1_YRPGTVALR (Nucleus); 6: GRP78_LTPEEIER (ER); 7: LAMP1_TVESITDIR (Lysosome); 8: HSP60_LSDGVAVLK (Mitochondria); 9: CYP3A4_EVTNFLR; 10: Rab7_VIILGDSGVGK (Late endosome); 11: LAMP1_VWVQAFK (Lysosome); 12: Rab7_ATIGADFLTK (Late endosome); 13: Rab5_LVLLGESAVGK (Early endosome); 14: LAMP1_AFSVNIFK (Lysosome); 15: HSC70_FEELNADLFR (Cytosol); 16: FcRn_LFLEAFK.

The newly developed UPLC-MRM methods for subcellular organelle markers and case study proteins were demonstrated using their synthetic SIL heavy signature peptides (Fig. 2). Sixteen peptides for a total of ten proteins were separated and detected over a 6-min LC retention time window. This developed method was used in the following subcellular organelle profiling analysis.

### Subcellular organelle marker profiling using immunoblot

**Figure 3:**
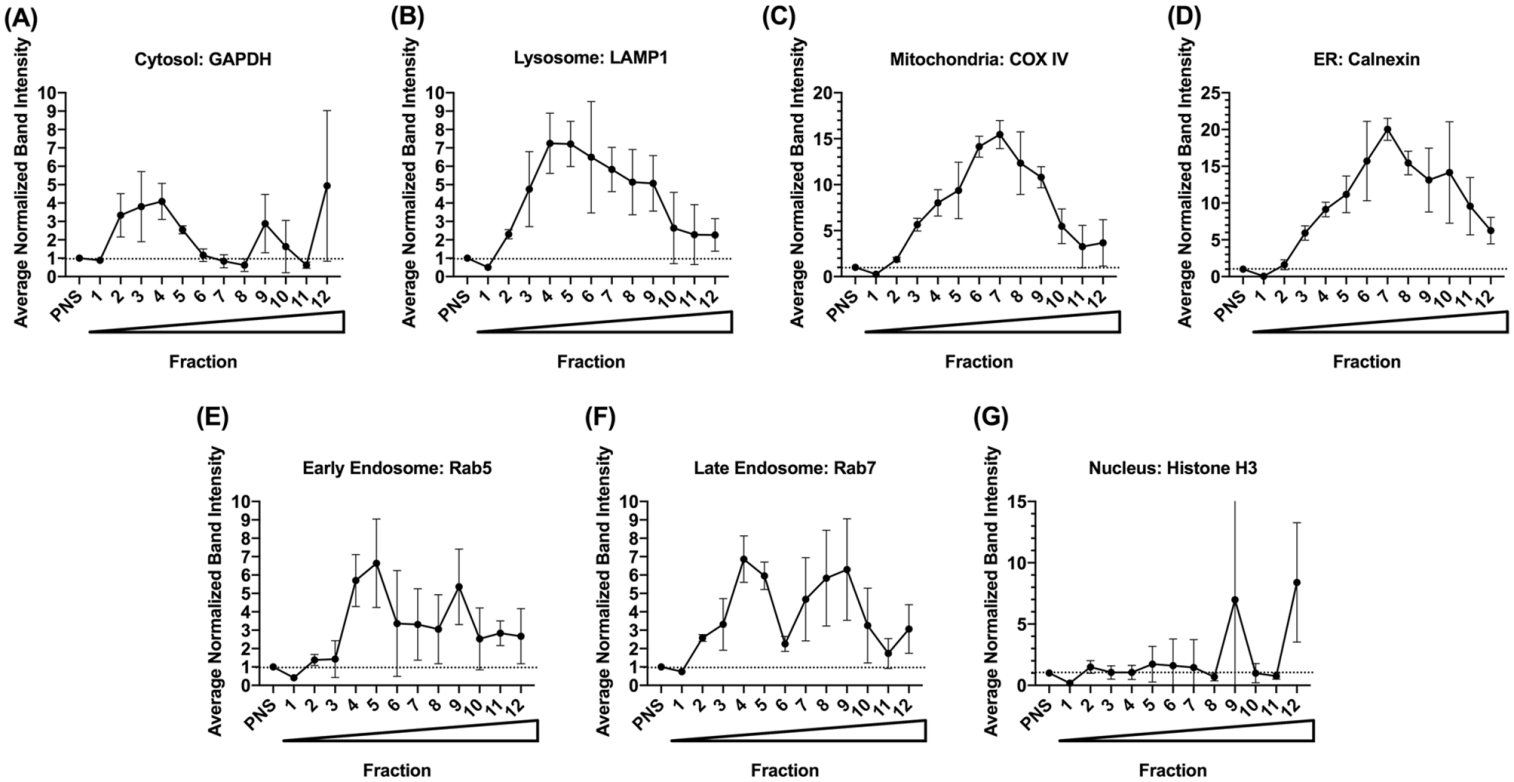
Subcellular organelle marker profiles characterized by immunoblot (normalized by protein amount loaded). All band intensities were normalized to per µg protein loaded, and each fraction’s band intensity was normalized to its corresponding PNS band intensity. Circles represent the average normalized band intensity by protein amount from three human liver donors, and error bars stand for the standard deviation. Wedge indicates increasing sucrose % from fraction 1 to 12.

Subcellular organelle markers were probed for each sucrose fraction and PNS collected from three human livers using immunoblot (Supplemental Figs. 1–3) and normalized results are presented in Fig. 3. The band intensity of each donor’s organelle marker was normalized to the total protein amount loaded onto the gel and then compared to the corresponding PNS band intensity. As such, a normalized band intensity value greater than 1 indicates an enrichment of the target protein in the sucrose fraction relative to the original PNS sample. Based on Fig. 3, early and late endosomes were most enriched in fractions 4, 5, and 9; lysosomes had a wider spread in the sucrose fractions, but peaked in fraction 4; both mitochondria and ER were mostly collected in fraction 7.

### Subcellular organelle marker profiling using UPLC-MRM-based targeted proteomics

**Figure 4:**
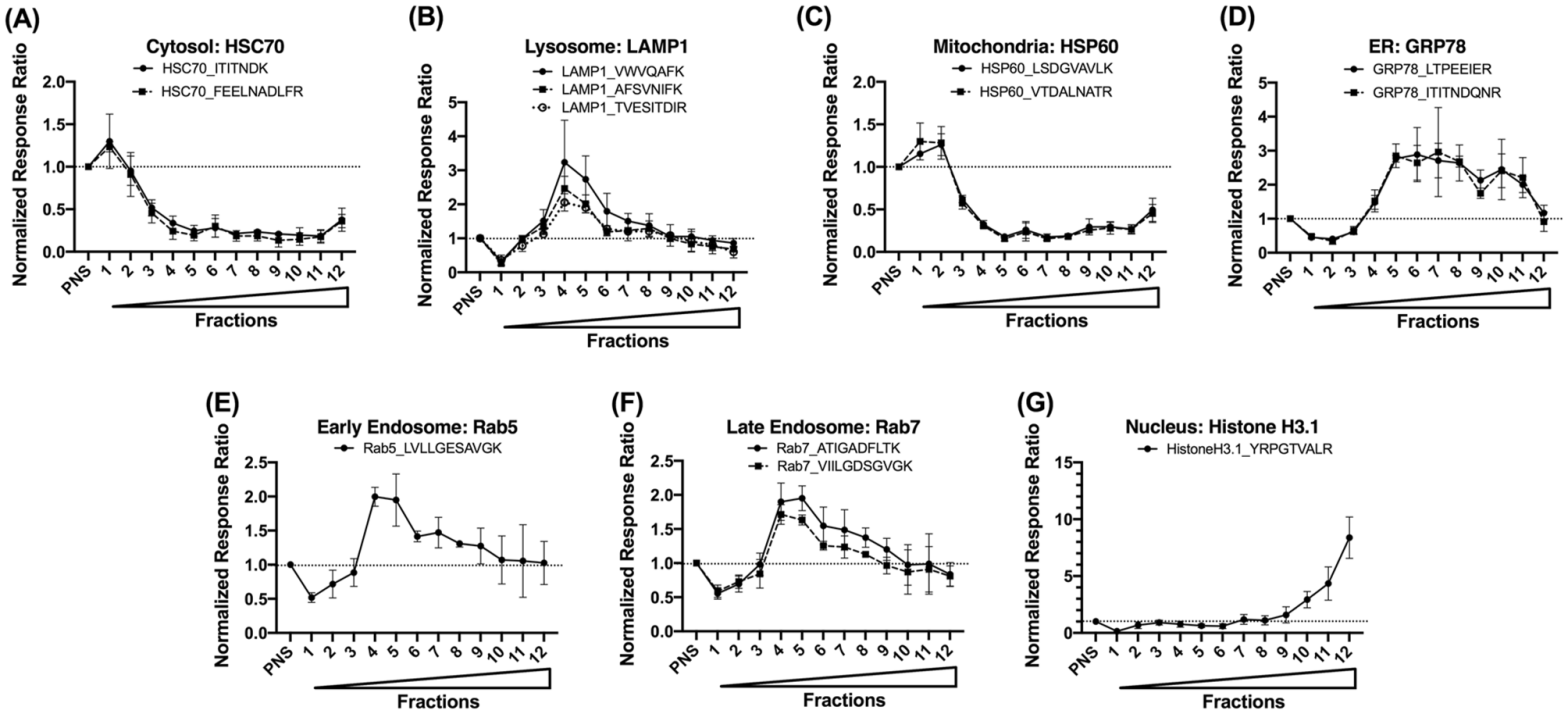
Subcellular organelle marker profiles characterized by UPLC-MRM. 20 µg total protein were digested for each sample. Relative quantification was achieved using response ratio, which corresponds to the ratio of light peptide area to spiked-in heavy labelled peptide area. Each fraction’s response ratio was normalized to its corresponding PNS response ratio. Solid circles, open circles, and squares represent the average normalized response ratio from three human liver donors, and error bars stand for the standard deviation. Wedge indicates increasing sucrose % from fraction 1 to 12.

A set of subcellular organelle markers was also profiled using the developed UPLC-MRM methods (Fig. 4). The raw data was included in the “Supplemental material”. Lysosomal marker, LAMP1, peaked in fraction 4, and both early and late endosomes were most abundant in fractions 4 and 5 based on their markers. In contrast, the ER marker GRP78 had a broader spread throughout the isolated fractions based on MRM analysis. Unexpectedly, the mitochondrial marker HSP60 was enriched in the top fractions 1 and 2 (see “Discussion” below).

### Distribution and recovery of human neonatal Fc receptor (FcRn), beta-2 microglobulin (β2M), and cytochrome P450 3A4 (CYP3A4) using UPLC-MRM-based targeted proteomics

**Figure 5:**
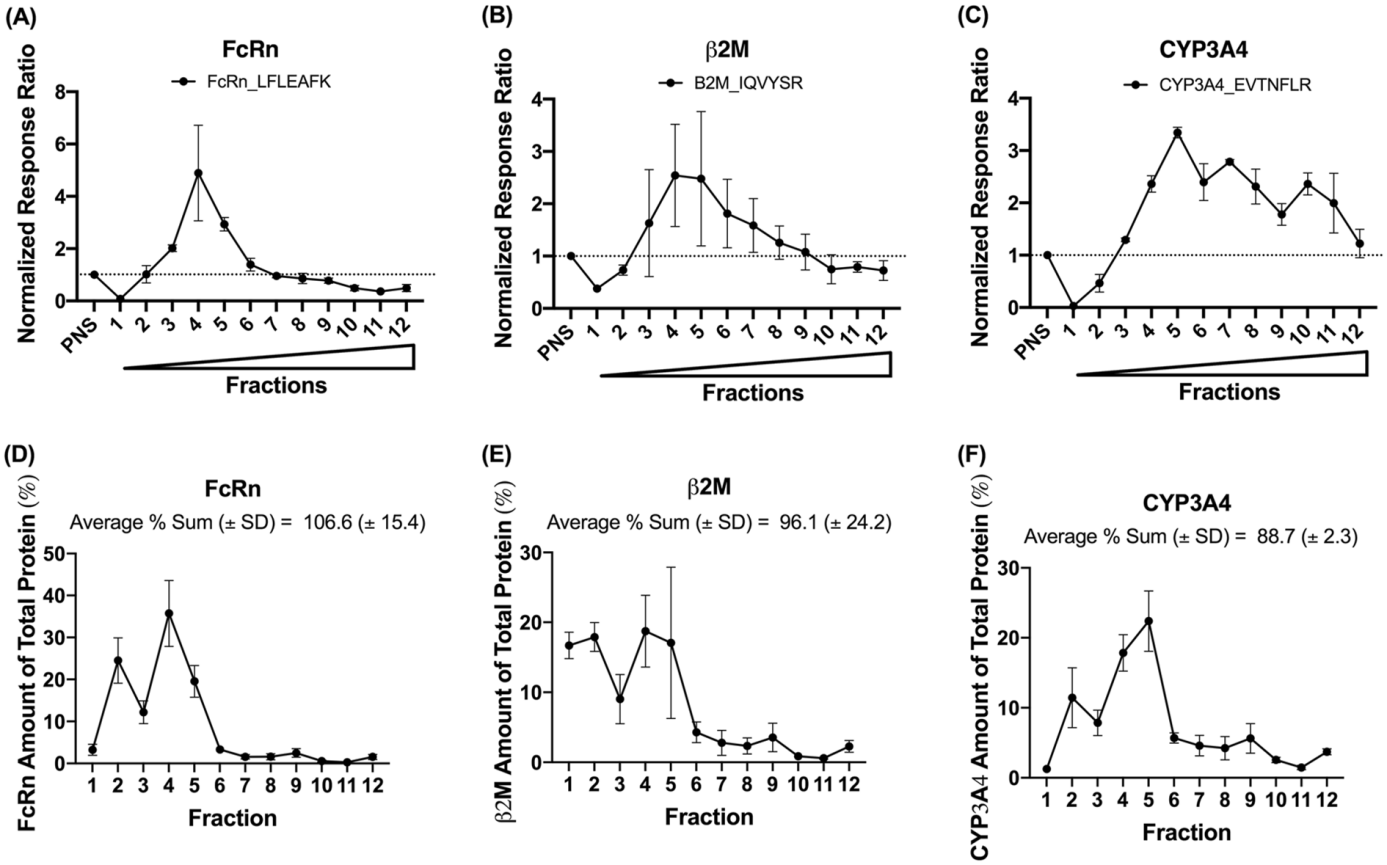
Distribution (**A**–**C**) and recovery (**D**–**F**) for three case study proteins in the isolated sucrose fractions. 20 µg of total protein were digested for each sample. Each fraction’s response ratio was normalized to its corresponding PNS response ratio. Circles represent the average normalized response ratio from three human liver donors, and error bars stand for the standard deviation. Wedge indicates increasing sucrose % from fraction 1 to 12.

Distribution and recovery of three case study proteins, FcRn, β2M, and CYP3A4, were plotted in Fig. 5. First, all response ratios were normalized to response ratio from respective PNS (Fig. 5A–C) to show enrichment. Both FcRn and β2M peaked in fraction 4, while CYP3A4 had a more widespread distribution. Then, recovery of each protein in each fraction (i.e., fraction of a protein present in each fraction relative to the total amount in PNS, Fig. 5D–F) was calculated as the following:

$$Recovery \%= \frac{{Normalized\,response\,ratio}_{fraction}*{[total\,protein]}_{fraction}*{Volume}_{fraction}}{{Normalized\,response\,ratio}_{PNS}*{[total \,protein]}_{PNS}*{Volume}_{PNS}}*100\%$$

Overall, all three proteins achieved excellent recovery from isolated fractions, with an average (± S.D.) of 106.6 (± 15.4) %, 96.1 (± 24.2) %, and 88.7 (± 2.3) % for FcRn, β2M, and CYP3A4, respectively.

## Discussion

Immunoblot has been the “gold standard” for assessing and characterizing the isolated subcellular organelles and requires a small amount (typically several μg of total protein) of samples for each gel lane. The procedure is very labor-intensive and time-consuming and requires antibodies for each targeted organelle marker that are either not accessible or of variable quality. Moreover, issues of specificity, narrow signal linear dynamic range, low reproducibility, and difficulty in multiplex ability render antibody-based immunoquantification (e.g., immunoblot) less than ideal for multiplexed protein detection and quantification^13^. Indeed, some of these issues were encountered in our immunoblot data (Fig. 3, Supplemental Figs. 1–3). Though each band intensity was normalized to its corresponding band signal in PNS to eliminate differential expression among different individuals, the error bars for immunoblot data points (42% CV, averaged from all the data points) were still much larger than the standard deviations from MRM data (21% CV, averaged from all the data points) (Figs. 3 and 4). The reduced variation from MRM-based targeted quantification is expected to improve robustness and reproducibility during quantification and characterization of targeted subcellular organelle marker proteins.

In addition, when compared to immunoblot method, UPLC-MRM-based methods also enable the possibility of multiplexed analysis, and hence, increase analysis throughput. In this study, all chromatograms of targeted organelle marker proteins and case study proteins from an individual fraction were acquired in a single 13.5-min LC-MRM run, which significantly reduced the sample analysis time. Furthermore, within the same run, multiple MRM transitions can be monitored for each signature peptide, and several signature peptides can be used as surrogates for each targeted protein. These measures can further improve the specificity of MRM analysis, which has already been conferred by carefully choosing protein-specific signature peptides and precursor-product ion pairs (Table 1). For organelle marker proteins with more than one signature tryptic peptide monitored, similar trend of protein distribution was observed using different signature peptides (Fig. 4), which demonstrates high consistency and specificity of the developed UPLC-MRM-based targeted proteomic method. Based on signal-to-noise (S/N) ratio, which was previously used to demonstrate MS instrumental sensitivity^14^, most of our signal has an S/N > 10 (“Supplemental material”), providing sufficient sensitivity for relative quantification analysis.

Overall, organelle marker characterizations obtained from the newly developed UPLC-MRM methods agree with those from immunoblot data in terms of distribution peak shape/trend with one exception (Supplemental Fig. 4): mitochondria markers exhibited different profiles using the two methods. For immunoblot analysis, a transmembrane mitochondria marker protein COX IV, was used, and it showed that mitochondria were most enriched in fraction 7 (Fig. 3C); in contrast, a soluble mitochondria marker protein, HSP60, was chosen for UPLC-MRM analysis, and indicated that mitochondria still remained in the top fractions, near where PNS was loaded (Fig. 4C). Such differential profiles may be explained by the disruption of organelles during tissue freezing and thawing cycles and during homogenization. As a result, these manipulations might have released the soluble HSP60 into the cytoplasm while the transmembrane COX IV stayed associated with the broken membranes of mitochondria. During the centrifugation, these broken membranes could reform into artificial organelle vesicles or hybrid-fusion vesicles^15^ or maintain as partial membrane pieces, and travel through the sucrose gradient, explaining transmembrane mitochondria marker COX IV’s appearance at fractions with higher sucrose content. In addition, even though HSP60 is generally considered as a mitochondria marker, HSP60 can also be expressed in other subcellular compartments in response to specific events (e.g., infection and other disease states)^16^, and will accumulate in the cytosol when apoptosis occurs^17^. Therefore, appropriate mitochondria markers should be chosen depending on whether the protein of interest is a soluble or transmembrane protein, and the disease state of donor tissues or cell lines also need to be taken into consideration.

When selecting mitochondrial markers for this study, different criteria were considered for marker selection between immunoblot and UPLC-MRM. For immunoblot, the availability of specific antibodies and antibody specificity were the top priorities to consider in terms of marker selection, and membrane-bound or transmembrane markers were chosen to track reformed particles during mechanical disruption and centrifugation. However, for UPLC-MRM, soluble organelle protein markers were favored as transmembrane proteins tend to suffer solubility issues during sample preparation and are harder to digest using trypsin. Therefore, as a small transmembrane protein, COX IV (19.6 kDa) was not considered a suitable mitochondria marker for the UPLC-MRM study, and HSP60 was chosen instead as it was a well-established mitochondria marker. For future studies, an alternative choice of a mitochondrial protein marker for UPLC-MRM analysis could be ATP synthase F_1_ subunit beta^5^, which is the soluble part of the mitochondria ATP synthase complex. Similar considerations should be taken in all subcellular organelle marker protein selection based on the platform of choice and the nature of analyte needs to be tracked.

Although it would be preferrable in theory to use the same panel of subcellular organelle marker proteins when comparing UPLC-MRM-based method to immunoblot method, additional factors and limitations have to be considered in practice. In this study, the same lysosome, early endosome, late endosome and nucleus marker proteins were used for both immunoblot and UPLC-MRM methods, which provides a direct “head-to-head” comparison between the two methods. In contrast, different mitochondria markers COX IV and HSP60, cytosol markers GAPDH and HSC70, and ER markers calnexin and GRP78 were selected for immunoblot and UPLC-MRM methods, respectively, due to limited availability of quality antibodies and whether marker proteins were amenable to UPLC-MRM method development, e.g., protein size, transmembrane nature and signature peptide availability. It should also be noted that all selected proteins have been considered well-known markers to their respective subcellular organelle^18–20^ and should track subcellular distribution as expected. Indeed, ER and cytosol markers showed similar trends between the two methods (except for mitochondria markers as discussed above), with ER markers peaking in fraction 7 and cytosol markers peaking in top fractions (Figs. 3 and 4). However, our results also revealed that additional attention should be paid to marker protein selection, requiring customization depending on study purpose (in situ vs. fractionation), analysis methods used (immunoblot vs. LC/MS-based proteomics), and marker proteins themselves (membrane-bound vs. soluble).

Three case study proteins, human neonatal FcRn, β2M, and CYP3A4, were monitored to determine their distribution in this sucrose gradient. FcRn and β2M are binding partners, and bind to immunoglobulin G and albumin when in complex under acidic pH in the endosomal compartments^7^. Both FcRn and β2M were most enriched in fractions 4 and 5, and thus showed a similar pattern as the two endosomal markers, Rab5 and Rab7 (Figs. 4E,F and 5A,B). CYP3A4 is an ER protein that is responsible for the metabolism of many xenobiotics in the human liver. Its distribution mirrored the ER marker protein GRP78 (Figs. 4D and 5C). These results demonstrated that the developed organelle marker panel and the UPLC-MRM methods can be used as an alternative method to track the distribution of proteins of interest within the same injection for organelle quality assessment, especially when specific antibodies are unavailable commercially.

Based on our UPLC-MRM data, all marker proteins and example proteins were enriched in isolated organelle fractions with respect to originally loaded PNS signal (Figs. 4 and 5). Such enhancement allows subcellular fractionation using a sucrose gradient to be potentially used as an enrichment method in sample preparations in bioanalysis to increase the detection of low abundance proteins and biomarkers. However, one of the foreseeable problems would be the matrix difference among different sucrose fractions, as variabilities in matrices may potentially lead to differential ionization efficiencies of the same target by LC–MS-based methods. Therefore, parallelism^21^ and matrix effects should be carefully assessed if sucrose fractionation will be used as the sample preparation strategy. There is also a discrepancy in enrichment ratios presented in Figs. 3 and 4 (y-axis), and this might be due to the differences in detection methods. We believe that our LC–MS method gave a more accurate picture of enrichment than immunoblot due to great (almost 100%) recovery based on MS quantification (Fig. 5).

In conclusion, this study developed a UPLC-MRM method as an alternative means to traditional immunoblot analysis for subcellular organelle marker protein characterization and profiling. The developed UPLC-MRM method was demonstrated to trace the migration profile of various organelles by following selected marker proteins, and to determine the distribution and recovery of target proteins. It is expected that this new method will facilitate multiplexed subcellular organelle quality assessment, organelle marker protein standardization, and intracellular drug disposition analysis. It was also demonstrated that sucrose gradient fractionation enriched specific target proteins based on their localization in subcellular organelles, which will aid future sample enrichment during bioanalysis of low abundance proteins and biomarkers.

## Supplementary Information

Supplementary Information 1.

Supplementary Information 2.

Supplementary Information 3.
