# Supplementary material for: Development of a UPLC-MRM-based targeted proteomic method to profile subcellular organelle marker proteins from human liver tissues
Source: Sci Rep. 2022 Jun 29;12:10985. doi: 10.1038/s41598-022-15171-0 (PMC9243099; doi:10.1038/s41598-022-15171-0)
Supplement: Supplementary file 2 — Supplementary Information 2. [file 41598_2022_15171_MOESM2_ESM.docx]

**Supplemental Data**

**Development of a UPLC-MRM-Based Targeted Proteomic Method to Profile Subcellular Organelle Marker Proteins from Human Liver Tissues**

Xiazi Qiu, Laura M. Doyle, and Michael Zhuo Wang

Department of Pharmaceutical Chemistry, School of Pharmacy, University of Kansas, Lawrence, Kansas, USA

**Supplemental Table 1.** Summary of human liver donor information (n = 3).

| **University of Kansas Medical Center Liver Bank** | | | | |  | | |  |
| --- | --- | --- | --- | --- | --- | --- | --- | --- |
| **KULTBID** | **Tube** | **Procedure Type** | **Pathological Diagnostics** | **Pathological Comments** | | **Age** | **Sex** | **Race** |
| L1017D | 1 | Transplant-Donor | No diagnostic abnormalities | Anoxia (Cardiovascular); Post-perfusion biopsy | | 30 | F | Caucasian |
| L369D | 1 | Transplant-Donor | No diagnostic abnormalities | Gunshot wound (Head) | | 33 | M | Caucasian |
| L791D | 1 | Transplant-Donor | No diagnostic abnormalities | Anoxia (Cardiovascular) | | 53 | M | Caucasian |

**Supplemental Table 2.** Summary of antibody information used in immunoblot analysis.

| Target and Antibody Name | | Dilution used | Company/Source | Cat. no. |
| --- | --- | --- | --- | --- |
| Lysosome: LAMP1 | LAMP1 (D2D11) XP® Rabbit mAb | 1/2000 in 5% (w/v) BSA in 1X TBST 0.1% | Cell Signaling Technology | 9091 |
| Nucleus: Histone H3 | Histone H3 (D1H2) XP® Rabbit mAb | 1/1000 in 5% (w/v) milk in 1X TBST 0.1% | Cell Signaling Technology | 4499 |
| Early Endosome: Rab5 | Rab5 (C8B1) Rabbit mAb | 1/1000 in 5% (w/v) BSA in 1X TBST 0.1% | Cell Signaling Technology | 3547 |
| Late Endosome: Rab7 | Rab7 (D95F2) XP® Rabbit mAb | 1/1000 in 5% (w/v) BSA in 1X TBST 0.1% | Cell Signaling Technology | 9367 |
| Mitochondria: COX IV | COX IV (3E11) Rabbit mAb | 1/5000 in 5% (w/v) BSA in 1X TBST 0.1% | Cell Signaling Technology | 4850 |
| ER membrane: Calnexin | Calnexin (C5C9) Rabbit mAb | 1/1000 in 5% (w/v) BSA in 1X TBST 0.1% | Cell Signaling Technology | 2679 |
| Cytosol: GAPDH | GAPDH (14C10) Rabbit mAb | 1/5000 in 5% (w/v) BSA in 1X TBST 0.1% | Cell Signaling Technology | 2118 |
| Secondary Antibody | Goat Anti-rabbit IgG, HRP-linked Antibody | 1/10000 in 1X TBST 0.1% | Cell Signaling Technology | 7074 |


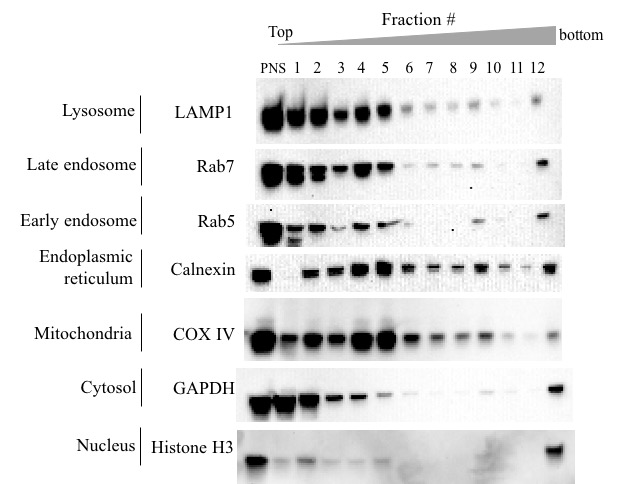


**Supplemental Figure 1. Subcellular organelle marker profiles for Donor L791D1 characterized by immunoblot.**

**
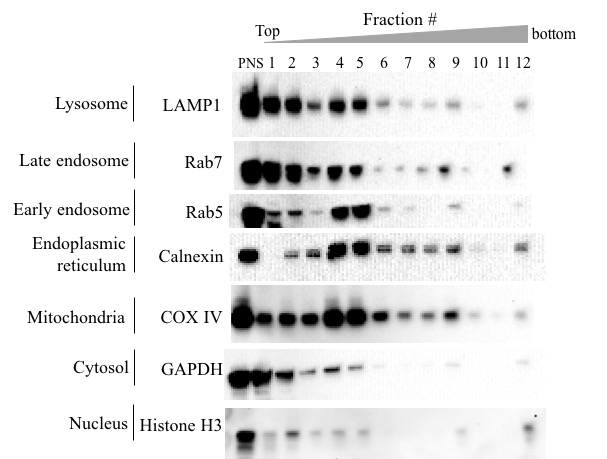
**

**Supplemental Figure 2. Subcellular organelle marker profiles for Donor L369D1 characterized by immunoblot.**

**
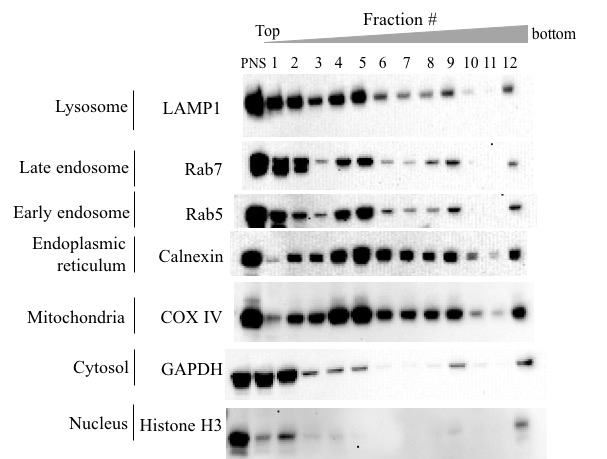
**

**Supplemental Figure 3. Subcellular organelle marker profiles for Donor L1017D1 characterized by immunoblot.**


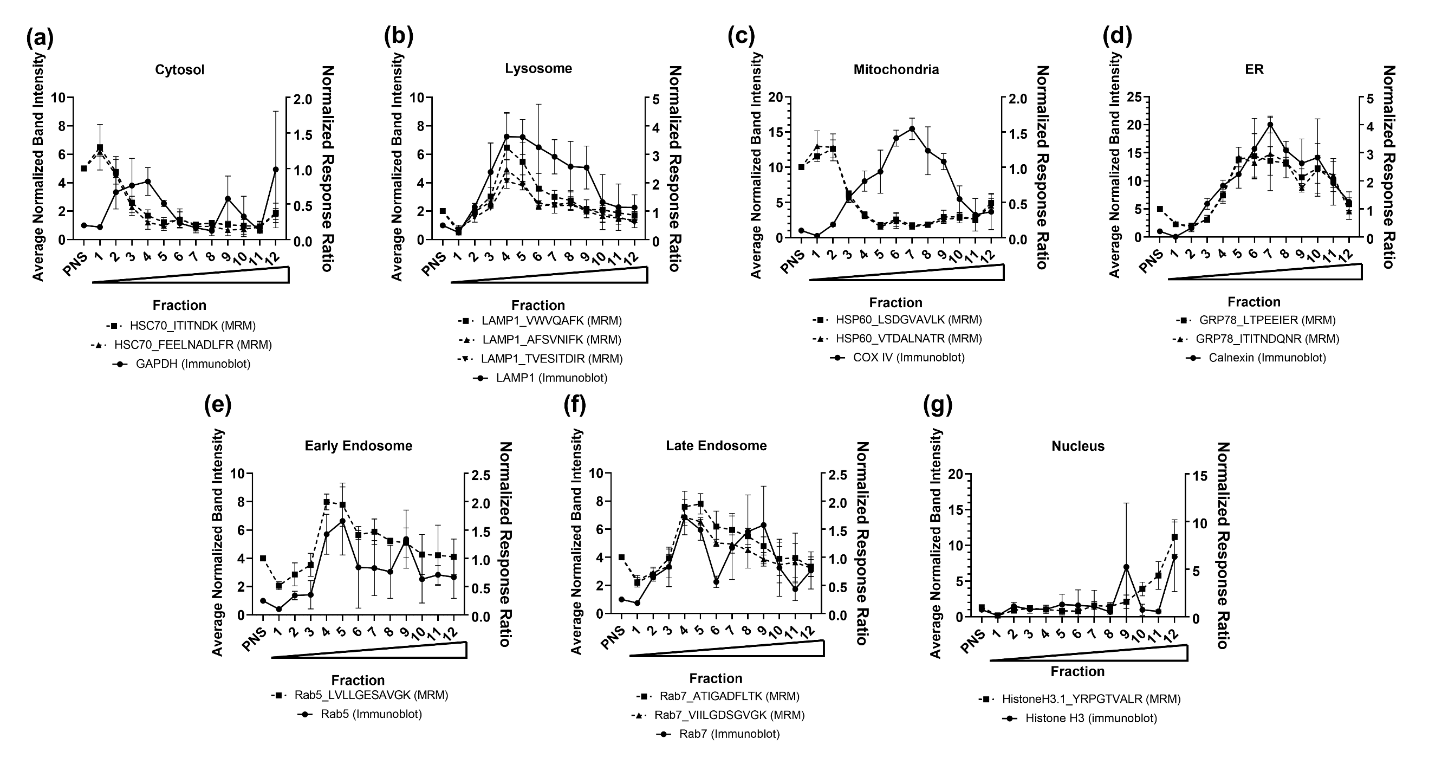


**Supplemental Figure 4. Overlayed subcellular organelle marker profiles characterized by UPLC-MRM and immunoblots.** Data from MRM methods were plotted on the right y-axis. 20 µg total protein were digested for each sample. Relative quantification was achieved using response ratio, which corresponds to the ratio of light peptide area to spiked-in heavy labelled peptide area. Each fraction’s response ratio was normalized to its corresponding PNS response ratio. Squares and/or triangles represent the average normalized response ratio from three human liver donors, and error bars stand for the standard deviation. Data from immunoblot methods were plotted on the left y-axis. All band intensities were normalized to per µg protein loaded, and each fraction’s band intensity was normalized to its corresponding PNS band intensity. Circles represent the average normalized band intensity by protein amount from three human liver donors, and error bars stand for the standard deviation. Wedge indicates increasing sucrose % from fraction 1 to 12.
