# Supplementary material for: Development of a UPLC-MRM-based targeted proteomic method to profile subcellular organelle marker proteins from human liver tissues
Source: Sci Rep. 2022 Jun 29;12:10985. doi: 10.1038/s41598-022-15171-0 (PMC9243099; doi:10.1038/s41598-022-15171-0)

Calnexin

Overlay of ladder and blot to show edges of the blots

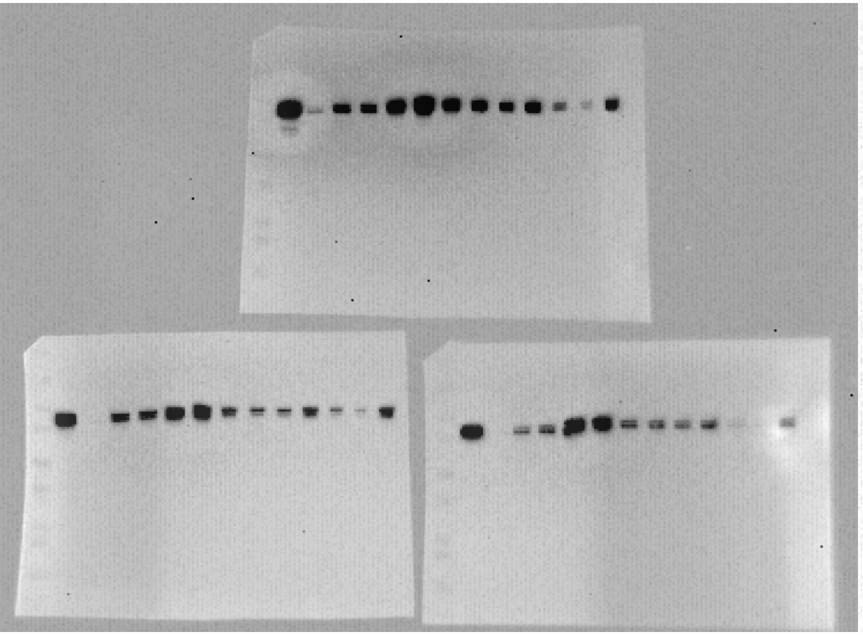

Blots only

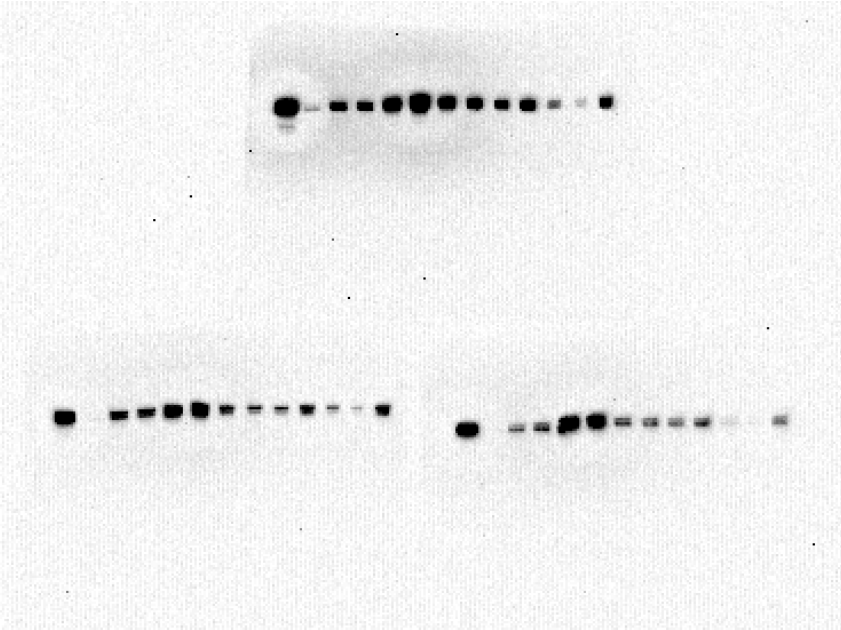

Ladders

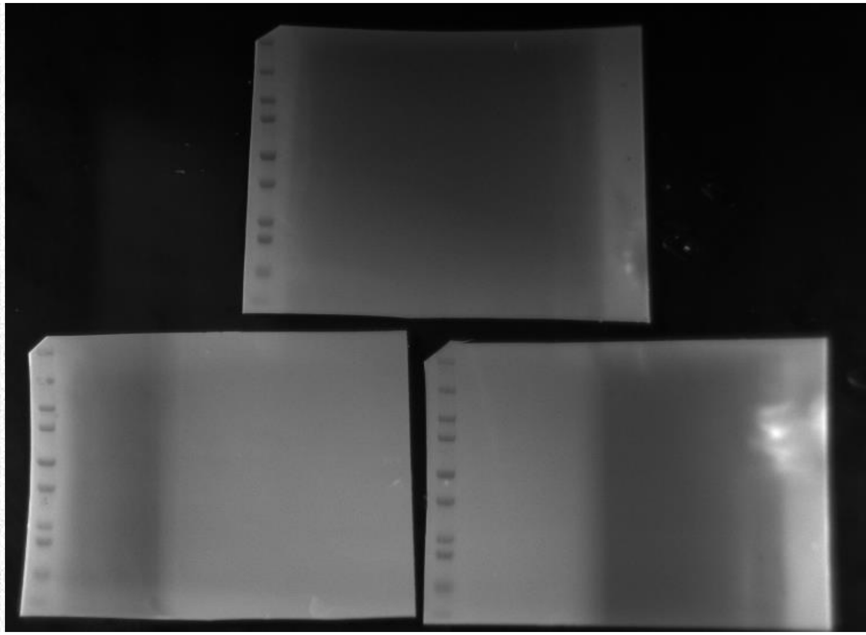

COX IV

Overlay of ladder and blot to show edges of the blots

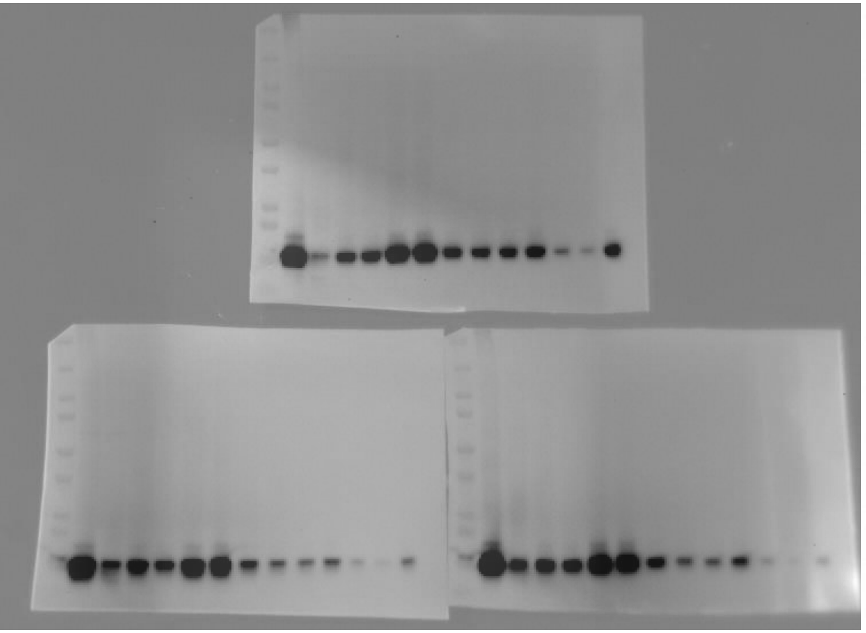

Blots only

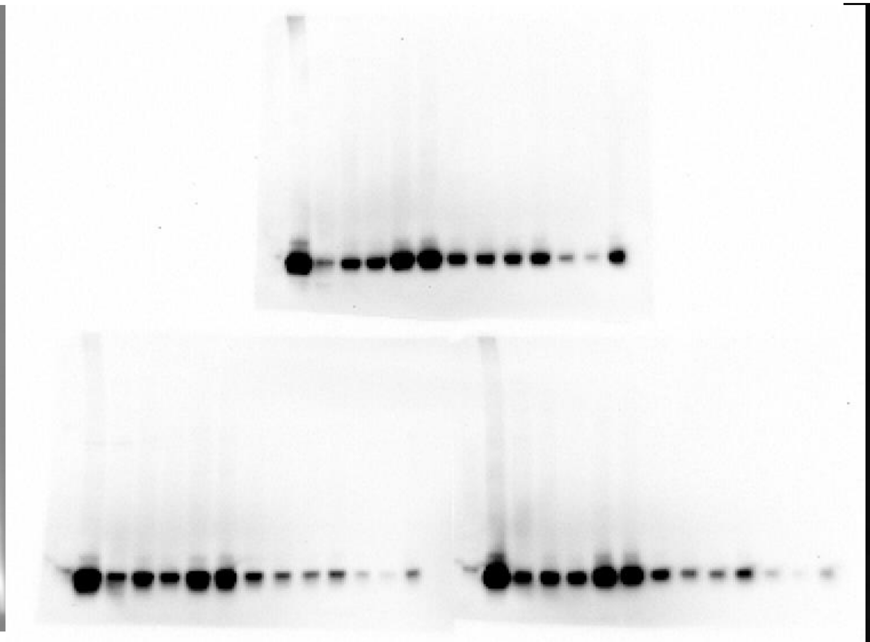

Ladders

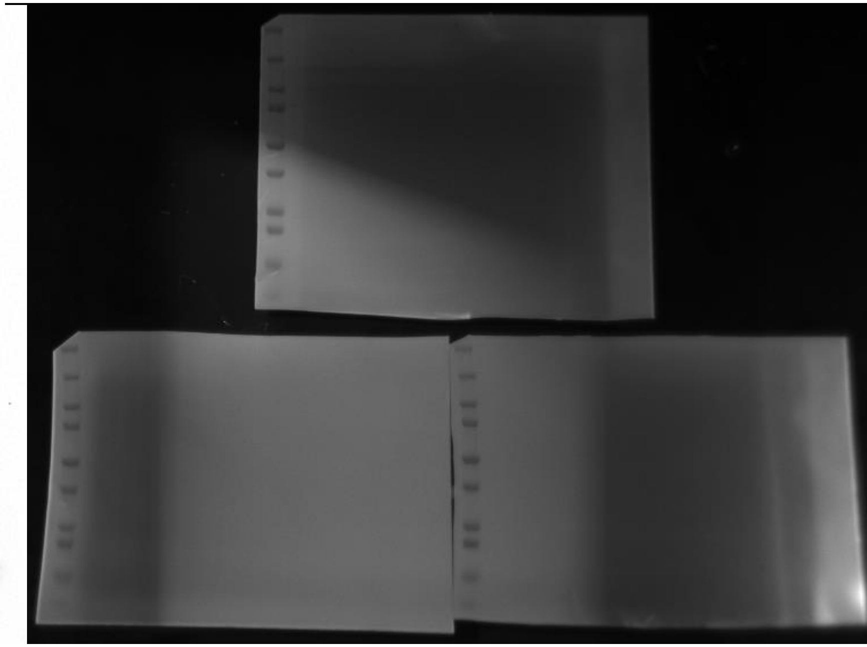

GAPDH

Overlay of ladder and blot to show edges of the blots

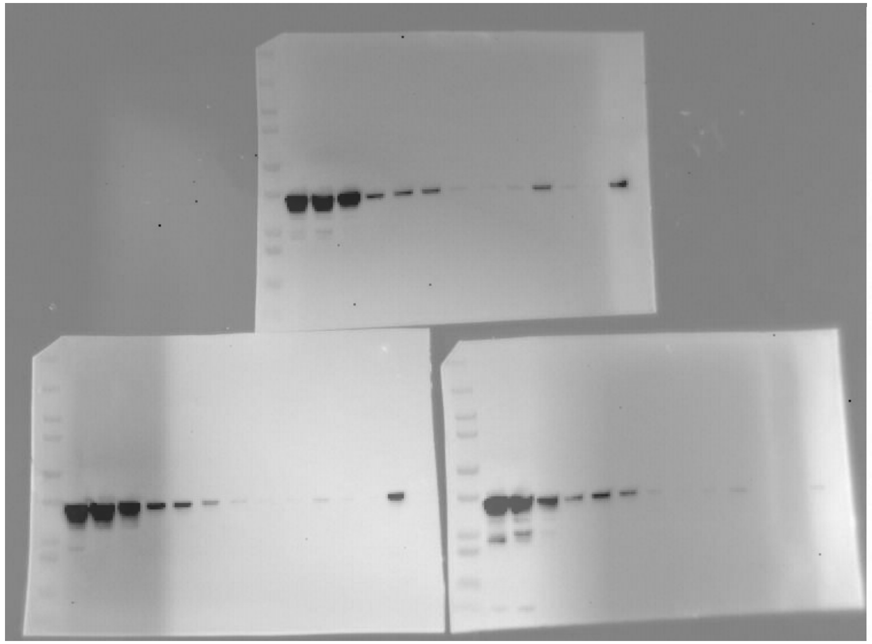

Blots only

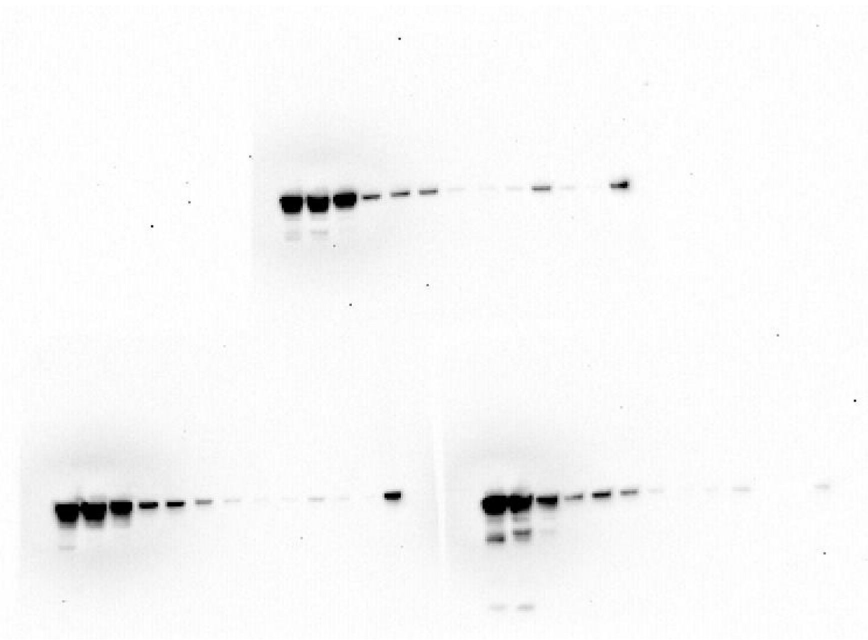

Ladders

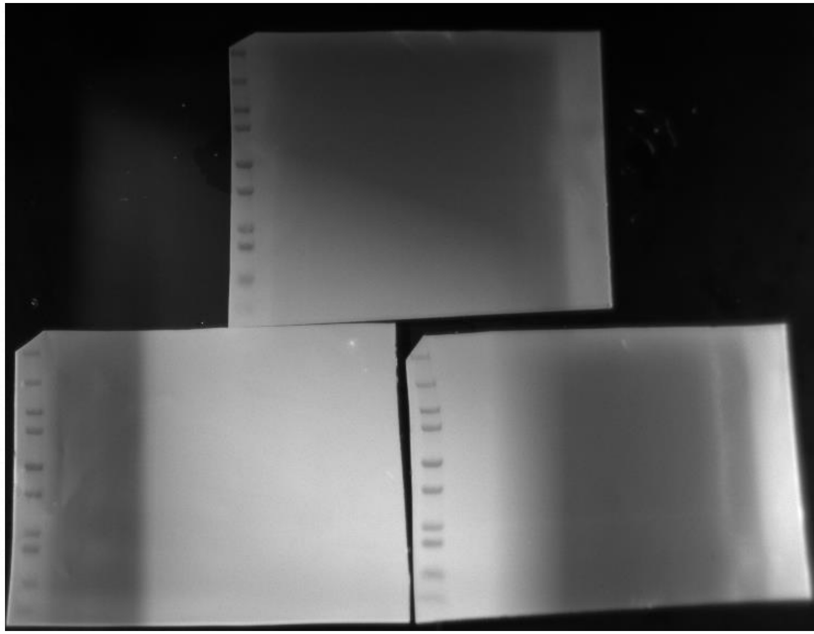

Histone H3

Overlay of ladder and blot to
show edges of the blots

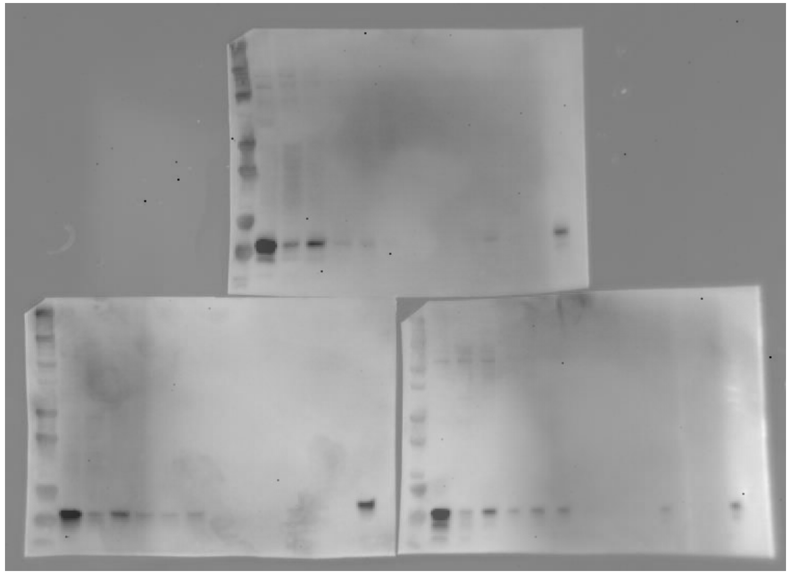

Blots only

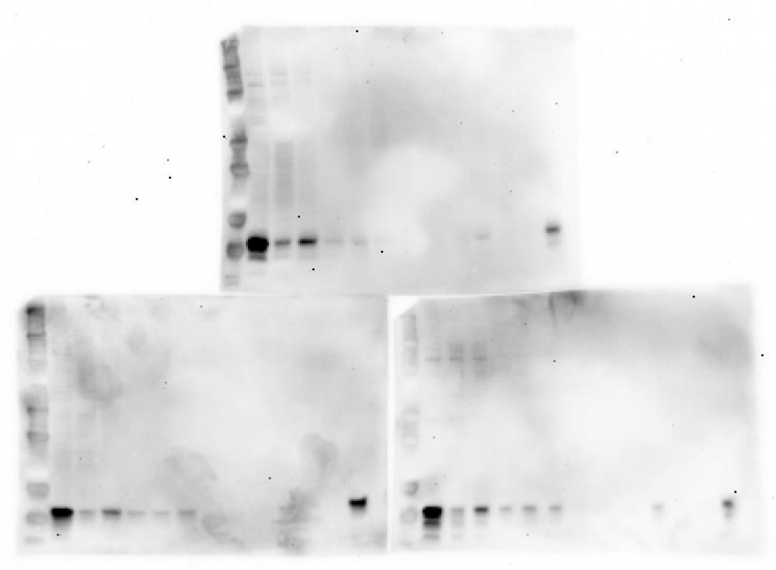

Ladders

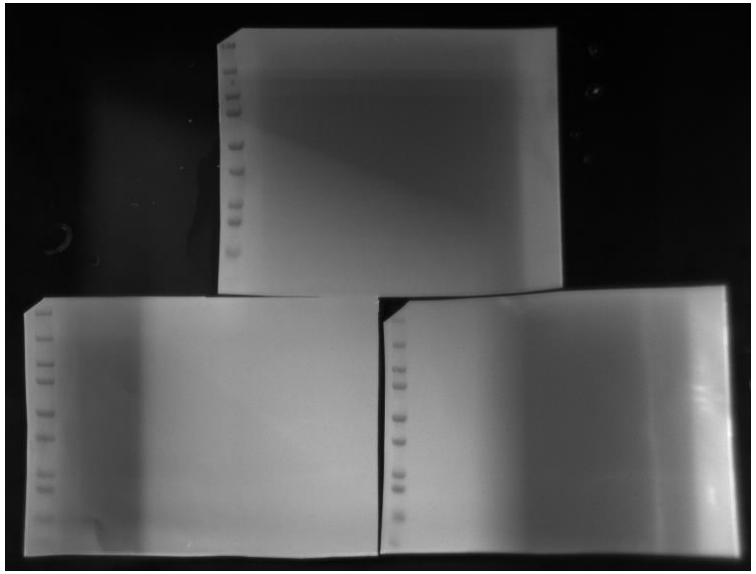

LAMP1

Overlay of ladder and blot to show edges of the blots

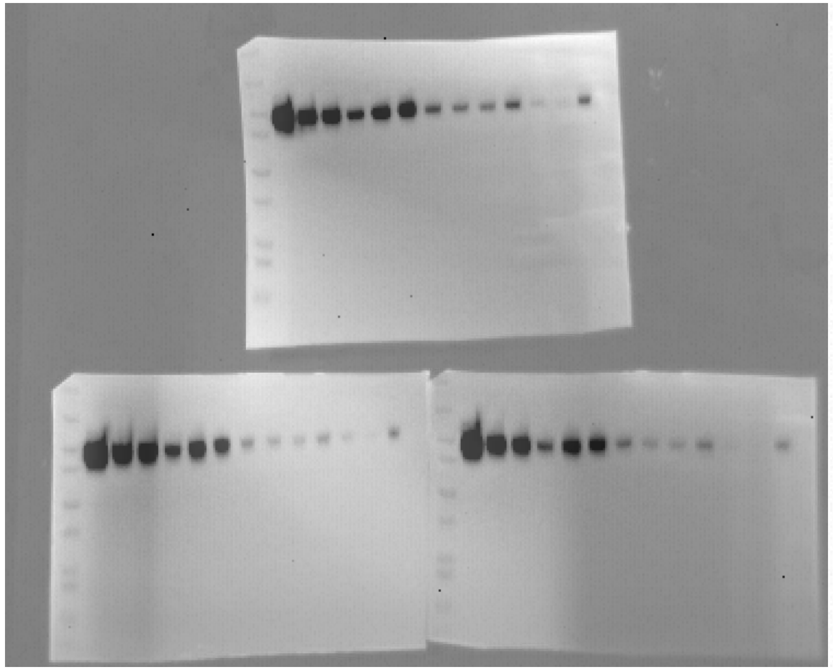

Blots only

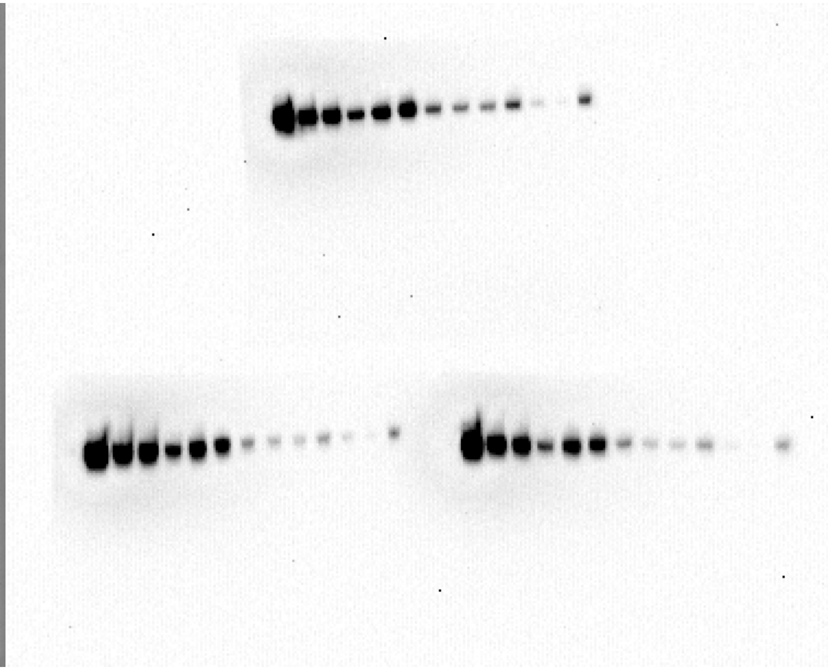

Ladders

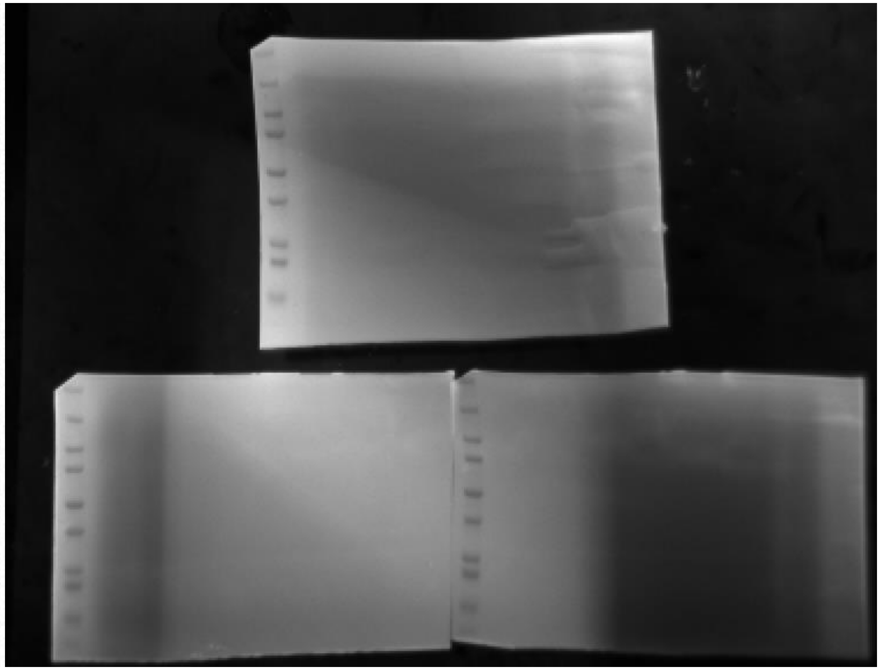

Rab5

Overlay of ladder and blot to show edges of the blots

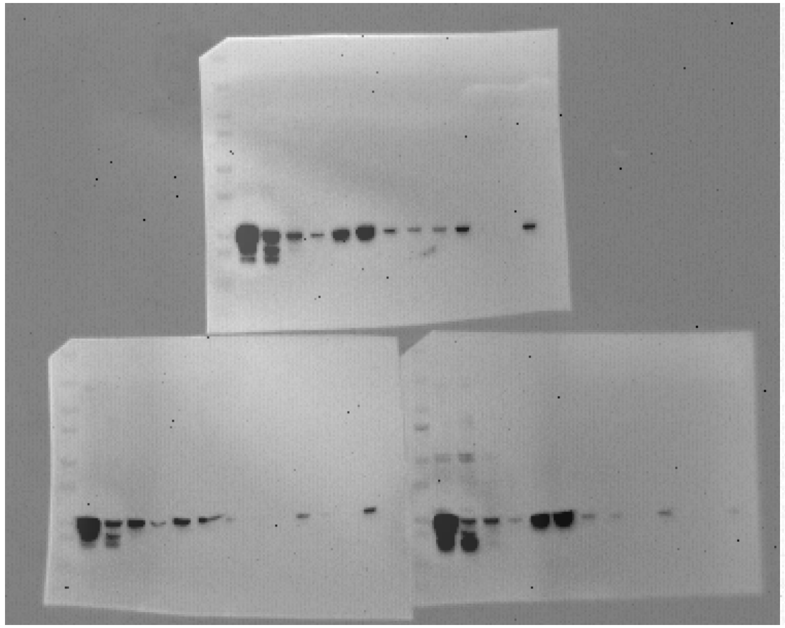

Blots only

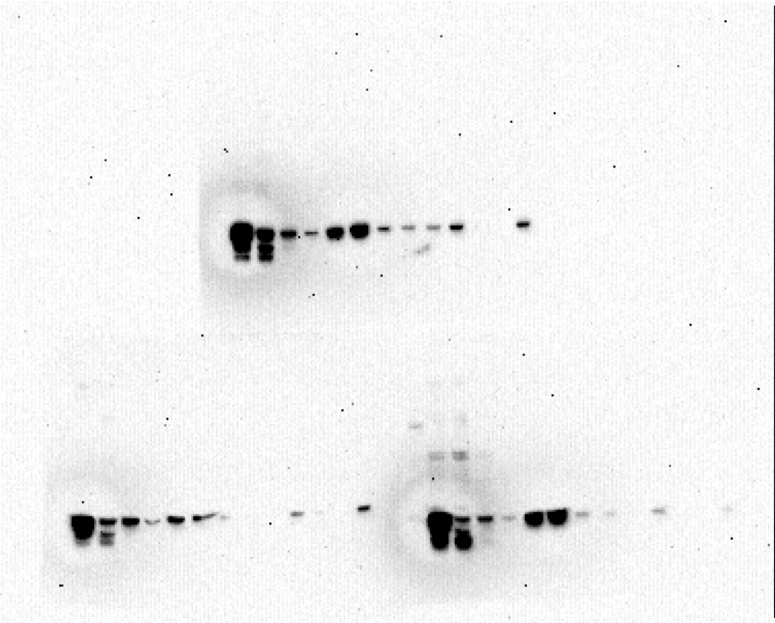

Ladders

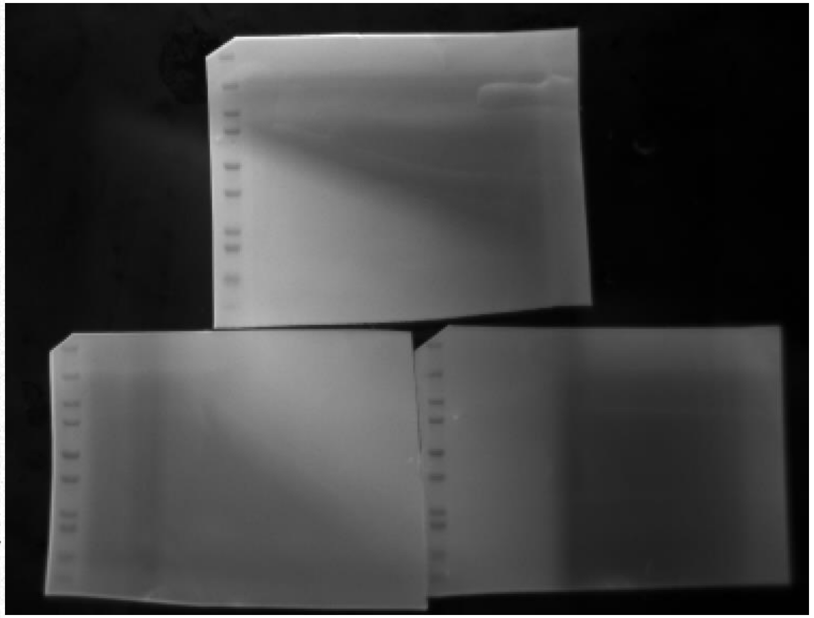

Rab7

Overlay of ladder and blot to show edges of the blots

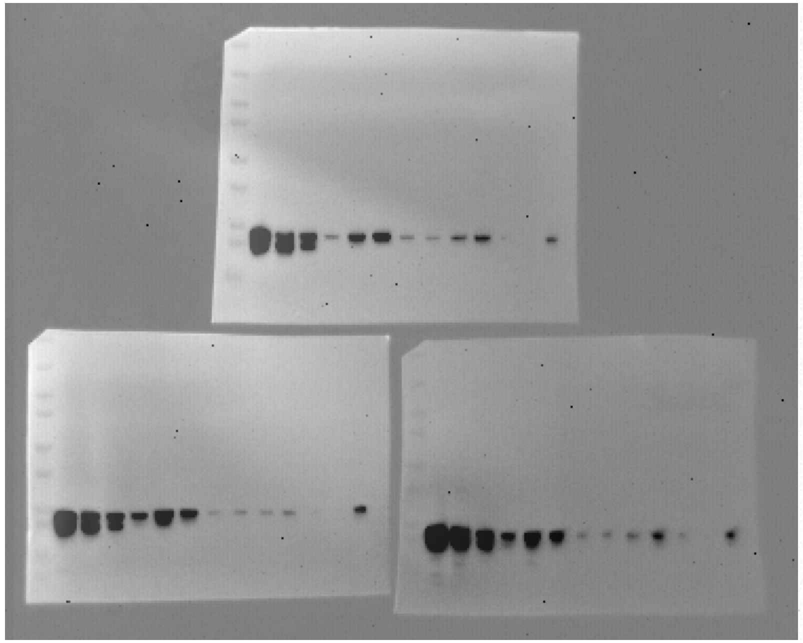

Blots only

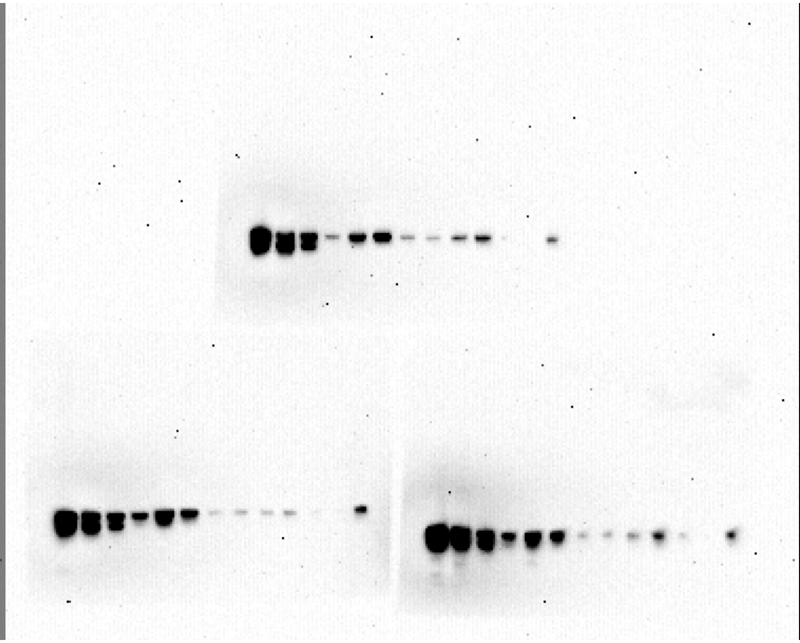

Ladders

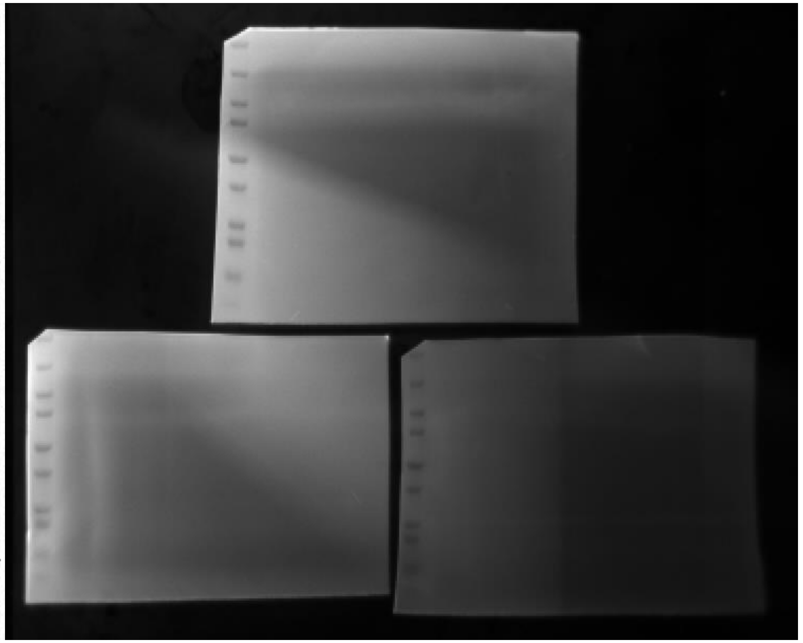

Supplement: Supplementary file 3 — Supplementary Information 3. [file 41598_2022_15171_MOESM3_ESM.pdf]
